# Introducing Typhoid Conjugate Vaccine in South Asia: Lessons From the Surveillance for Enteric Fever in Asia Project

**DOI:** 10.1093/cid/ciaa1296

**Published:** 2020-12-01

**Authors:** Alice S Carter, Stephen P Luby, Denise O Garrett

**Affiliations:** 1 Applied Epidemiology, Sabin Vaccine Institute, Washington, DC, USA; 2 Division of Infectious Diseases and Geographic Medicine, Stanford University School of Medicine, Stanford, California, USA

**Keywords:** typhoid, paratyphoid, enteric fever, *Salmonella* Typhi, surveillance

## Abstract

Enteric fever remains a public health concern in communities lacking sanitation infrastructure to separate sewage from drinking water. To bridge the gap until large-scale civil-engineering projects are implemented in high-burden countries, typhoid conjugate vaccine presents a promising disease-prevention technology. A new typhoid conjugate vaccine was prequalified by the World Health Organization in 2017 and is beginning to be introduced in countries around the world. To help inform vaccine introduction, the Surveillance for Enteric Fever in Asia Project (SEAP) conducts prospective enteric fever surveillance in Bangladesh, Nepal, and Pakistan. This supplement presents findings from Phase II of the study (2016–2019) on burden of disease, clinical presentation, the growing spread of drug-resistant strains, and policy and economic ramifications. These findings are delivered to support policymakers in their deliberations on strategies to introduce typhoid conjugate vaccine as a preventive tool against enteric fever.

Enteric fever, caused by *Salmonella enterica* subspecies *enterica* serovars Typhi and Paratyphi A, has been controlled in settings with sanitation infrastructure that reliably separates human excrement from the community’s water and food supply. In settings that lack these capacities, the burden of enteric fever has been managed for decades by treatment with low-cost antibiotics. However, the spread of drug resistance undermines this disease-management approach [1, 2]. Typhoid conjugate vaccine (TCV), prequalified by the World Health Organization (WHO) in 2017, presents a promising preventive tool that may lessen the burden of disease while governments implement more definitive disease-prevention approaches.

Accurate estimates of not only disease incidence but also severity, cost of illness, and other disease-burden metrics can help policymakers make informed vaccine-introduction decisions. Disease surveillance can provide this information. Early research, such as the Diseases of the Most Impoverished Program (2000–2006), found wide variability in enteric fever incidence across different Asian countries [3]. However, existing research had limited findings on severity and complications. With funding from the Bill & Melinda Gates Foundation, the Sabin Vaccine Institute initiated the Surveillance for Enteric Fever in Asia Project (SEAP) in 2014 to fill knowledge gaps in the incidence, severity, and cost of enteric fever in Bangladesh, Nepal, and Pakistan. Similar surveillance efforts were launched through the Typhoid Surveillance in Africa Program (later succeeded by Severe Typhoid Fever in Africa [SETA]) and by Severe Enteric Fever in India (SEFI).

The SEAP team consists of partners affiliated with the Sabin Vaccine Institute, the US Centers for Disease Control and Prevention, Stanford University, Aga Khan University in Pakistan, the Child Health Research Foundation in Bangladesh, and Dhulikhel Hospital in Nepal. In the first phase of SEAP, the research team conducted retrospective record reviews, identifying and mapping communities with frequent typhoid infections. As a result of this work, a 12-paper supplement was published in 2018 [4]. Building on this foundational research, Phase II was conducted from September 2016 through September 2019, combining prospective, hospital-based surveillance at 6 study sites with community-based healthcare utilization surveys to generate incidence estimates of enteric fever.

This supplement presents findings from 3 years of SEAP’s prospective enteric fever surveillance in Bangladesh, Nepal, and Pakistan, with a focus on clinical presentation, disease severity, the rise of antimicrobial resistance (AMR), and the cost of illness to patients, caregivers, and healthcare providers.

## THE SURVEILLANCE FOR ENTERIC FEVER IN ASIA PROJECT

**Figure 1.**
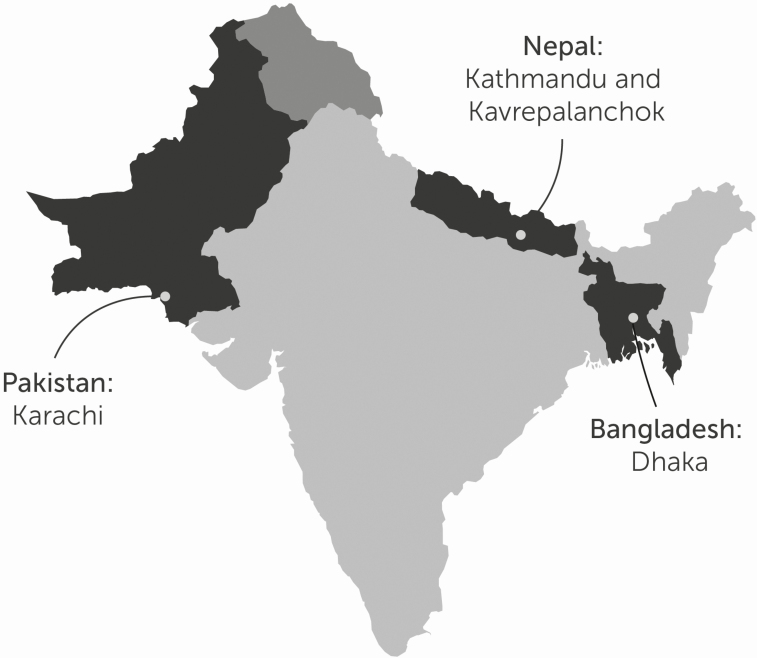
Locations of SEAP enteric fever surveillance catchment areas. Abbreviation: SEAP, Surveillance for Enteric Fever in Asia Project.

The SEAP team has built a robust infrastructure for enteric fever surveillance in Bangladesh, Nepal, and Pakistan. SEAP utilized a hybrid surveillance approach of combining clinic-based surveillance with healthcare utilization surveys to generate adjustment factors for incidence rate calculations. Using predefined residence catchment areas in Bangladesh, Nepal, and Pakistan (Figure 1), the team enrolled patients with fever at hospital outpatient and inpatient departments, as well as emergency and surgical wards, hospital laboratories, and laboratory network sites. Consenting patients with suspected enteric fever were enrolled in the study and observed while they were receiving care at the facility. Study personnel then administered a follow-up phone call 6 weeks later to obtain a report of patient outcomes.

In 2017–2018, the team conducted a healthcare utilization survey of the catchment areas to understand care-seeking behavior in the community. The healthcare utilization survey collected data on household demographics and assets and was used to generate adjustment factors for incidence estimates.

Community incidence rates were estimated through the application of adjustment factors, including sensitivity of blood culture, consent rates, care-seeking behavior, and differential care-seeking behavior between enteric fever cases and the broader population [5]. This hybrid approach to generating population-level incidence rates was less resource intensive than a cohort study and did not interfere with patient behavior, capturing a more accurate representation of care-seeking behavior and disease severity [6].

## RESULTS FROM 3 YEARS OF HYBRID SURVEILLANCE FOR ENTERIC FEVER

In this supplement, we have 2 manuscripts describing the healthcare utilization survey that measured community care-seeking behaviors. Yu et al [7] share details on the implementation of the survey, providing lessons for similar undertakings in other areas. Their manuscript describes the use of geographic information systems (GIS)–based geosurveys and field mapping to reduce the need to pre-enumerate households in the sampling area, simplifying the logistics for mass-scale community surveying. Andrews et al [8] describe the results of the healthcare utilization survey, which were used to estimate population incidence of enteric fever. Key findings include that children under the age of 5 were more likely than other ages to seek care at our study sites, and across all ages at least 1 marker of disease severity was positively associated with care seeking. Additionally, wealthier individuals were more likely to seek care at study hospitals, while poorer households were more likely to seek care at pharmacies. These findings confirm that most cases of typhoid fever are not detected by the healthcare system, and should be taken into account when generating incidence estimates.

Each site experienced the burden of typhoid differently. In Bangladesh, where the greatest burden of enteric fever was found in children younger than 5 years of age, 30% of patients with laboratory-confirmed enteric fever were hospitalized, and hospitalizations lasted a median of 7 days. As Saha et al [9] point out, this presents a sizable burden for hospitals and means that hospital beds are occupied by patients with preventable disease. In Nepal, where there have previously been few surveillance studies outside of 1 metropolitan area, Tamrakar et al describe how SEAP data show wide heterogeneity between urban, peri-urban, and rural settings. This heterogeneity should be considered when planning to introduce vaccine. In Pakistan, Yousafzai et al [10] note the burden of disease in children younger than 15 years of age and the rise in drug-resistant infections. The authors caution that the case-fatality rate could increase with the rise in antimicrobial resistance due to the observed increased complications in patients with extensively drug-resistant (XDR) typhoid.

The supplement addresses the disease burden factors of AMR, complications, and sequelae. The spread of AMR strains of *S*. Typhi within and beyond Pakistan is of serious concern. Qamar et al [11] show that, when measuring resistance to ampicillin, chloramphenicol, trimethoprim-sulphamethaxazole, fluoroquinolones, and ceftriaxone in Pakistan, a full 67% of strains exhibited multidrug resistance (MDR), and 53% were XDR. Extensively drug-resistant typhoid is resistant to first- and second-line treatment options, and azithromycin resistance has been identified in each of the study countries, pointing toward a future in which we may have organisms that do not reliably respond to oral antibiotics. Drug-resistance rates were lower in Bangladesh (17% MDR) and Nepal (2% MDR) but remain concerning as strains continue to circulate in the population. A substudy described by Vaidya et al found that 39% of enrolled patients who presented at the clinic with suspected enteric fever already had antibiotics in their urine, but that there was only modest correlation between reported and measured antibiotic use.

When examining complications and sequelae, study authors again found variability between different patient groups. While Longley et al [12] present findings of low death rate and complications when considering all patients with enteric fever, they also highlight differences in severity between outpatients and inpatients. For example, the case-fatality rate for inpatients was nearly 10 times higher than for outpatients, who typically presented with milder disease. Qazi et al note that nontraumatic intestinal perforation could be a serious but understudied complication. Despite the low death rate, patients across the sites experienced a 30% hospitalization rate and prolonged duration of hospitalization when admitted.

The supplement also investigates typhoid diagnostics using clinical features or blood culture. When looking at clinical features for diagnosis of enteric fever, Aiemjoy et al [13] show that, among evaluated features, absence of cough, fever at presentation, and being unable to complete normal activities for 3 or more days showed the highest specificity as diagnostic criteria. While these may serve as moderate predictors of enteric fever, when patients presented at the hospital with 3 or more days of fever, clinical features offered only limited diagnostic performance. Despite the limited value of relying on clinical features for diagnosis, Hemlock et al [14] also find that there is variability based on hospital site, age, and sex in whether the patient would be offered blood culture. The same paper finds that approximately 79% of enrolled patients were offered antibiotics empirically, with higher rates in patients with provisional diagnoses of enteric fever, lower respiratory tract infections, or urinary tract infections.

This supplement presents an analysis of the cost of illness of enteric fever to patients and caregivers and health facilities. Results suggest that the cost of a case of enteric fever is high in the 3 countries. In Bangladesh, the median direct medical and nonmedical costs for patients and caregivers per case of enteric fever were 152% of the country’s annual health expenditure per capita across the sample; in Nepal, they were equivalent to 70%; and in Pakistan, they were 341% (Mejia et al [15–17]). These new estimates of the cost of illness of enteric fever in 3 countries can improve the evaluation of the costs and benefits of enteric fever prevention and control measures, including TCVs, in similar settings.

## INFORMING TYPHOID VACCINE INTRODUCTION

The ultimate aim of generating high-quality burden-of-disease data is to help inform policymakers in their TCV introduction decisions. As Bangladesh, Nepal, and Pakistan make decisions about typhoid vaccine introduction timelines and strategies, SEAP is providing up-to-date incidence and disease-burden data.

As an example, in 2017, SEAP provided the data from more than 15 000 records of patients with enteric fever to the WHO that contributed, along with data from other sources, to the subsequent recommendation for TCV vaccination of infants and children over 6 months of age in typhoid-endemic countries [18]. Soon after updating its recommendation, WHO prequalified the first TCV [19]. SEAP data have also been shared with Gavi, the Vaccine Alliance, to inform decision making on vaccine introduction and have been used in-country to advocate for TCV introduction through Gavi, which announced a US$85 million funding window to support the introduction of TCVs [20].

Surveillance plays an important role in deciding who should be vaccinated, evaluating the impact of vaccine introduction, and monitoring the spread of AMR or replacement by Paratyphi A.

In 2019, the Pakistani government became the first to introduce TCV into their routine immunization schedule by launching with a mass vaccination campaign in Sindh Province. The vaccine-introduction decision was supported by data from Aga Khan University showing the spread of XDR typhoid. Other typhoid-endemic countries are now similarly tasked with the decision of whether and how to introduce the vaccine into their routine immunization schedule. Disaggregated data such as those from Nepal will be critical for health policymakers optimizing vaccine-introduction strategies. The SEAP, SETA, and SEFI surveillance projects are uniquely positioned to provide data to inform these decisions, as well as monitor disease-burden impacts of spreading drug resistance and track the impact of vaccine introduction.

Surveillance will play an important role in informing disease-control initiatives as drug resistance spreads. It will be critical to detect the spread of XDR strains into new populations and adjust prevention strategies and treatment options accordingly. We have already seen this take place in Pakistan, where quick detection and response led to the first use in an outbreak setting and the nationwide roll-out of TCV. Other countries in the region will likely follow suit. In addition to morbidity and mortality data, SEAP provides valuable information on the cost of illness to patients and healthcare systems, as well as on clinician behavior in diagnosing enteric fever and prescribing antibiotics.

It is not yet known whether the roll-out of the TCV will open a new niche for the spread of *S*. Paratyphi. *Salmonella* Typhi and *S.* Paratyphi A occupy similar niches in the environment and human body, so a forced reduction in *S*. Typhi following TCV introduction could increase the absolute fitness of *S*. Paratyphi A [21]. In the coming years, SEAP will enhance its surveillance for *S*. Paratyphi A to track changes in disease trends before and after vaccine introduction. A better understanding of the burden of disease of paratyphoid will also help inform the development and eventual introduction of combined vaccines against *S*. Typhi and *S*. Paratyphi A.

While the findings in this supplement can support vaccine-introduction decisions, they also highlight the need for improved diagnostics for enteric fever. Blood culture is the best-available diagnostic but suffers from low sensitivity, high cost, and multiple-day delay in the availability of results. The SEAP infrastructure is now being leveraged to conduct sero-epidemiological and environmental surveillance studies to validate new tools for broad population-based surveillance for enteric fever. There is also promising new work to validate an immunoglobulin A (IgA)–based rapid test that could be used in the clinical setting [22].

Determining how to target TCV introduction and then measuring its impact at national or subnational scales will require reliable estimates of disease burden across many contexts. SEAP will continue to generate incidence data necessary for policymakers to maintain comparability before and after TCV introduction for a robust impact evaluation.

## CONCLUSIONS

The SEAP data on typhoid and paratyphoid in South Asia fill a gap in understanding the local burden of disease and point the way forward for typhoid vaccine introduction. Continuing surveillance data will support policymakers in their vaccine-introduction decisions and help us monitor the impact of vaccine postintroduction.

## Notes

***Acknowledgments.*** The authors acknowledge all of the study staff and participants who contributed to this project.

***Financial support.*** This work was supported by a grant from the Bill and Melinda Gates Foundation (grant number INV-008335).

***Supplement sponsorship.*** This supplement is sponsored by the Sabin Vaccine Institute and made possible by a grant from the Bill & Melinda Gates Foundation.

***Potential conflicts of interest.*** The authors: No reported conflicts of interest. All authors have submitted the ICMJE Form for Disclosure of Potential Conflicts of Interest.
